# Transcriptome analysis of the model grass *Lolium temulentum* exposed to green leaf volatiles

**DOI:** 10.1186/s12870-019-1799-6

**Published:** 2019-05-28

**Authors:** James E. Dombrowski, Brent A. Kronmiller, Vicky G. Hollenbeck, Adelaide C. Rhodes, John A. Henning, Ruth C. Martin

**Affiliations:** 1 USDA-ARS, National Forage Seed Production Research Center, 3450 SW Campus Way, Corvallis, Oregon, 97331-7102 USA; 2 Center for Genome Research and Biocomputing, Oregon State University, Corvallis, OR 97331 USA

**Keywords:** Grass, Green leaf volatiles, JA, *Lolium*, Priming, Signaling, Transcriptome, VOC, Wounding

## Abstract

**Background:**

Forage and turf grasses are routinely cut and grazed upon throughout their lifecycle. When grasses are cut or damaged, they rapidly release a volatile chemical cocktail called green leaf volatiles (GLV). Previously we have shown that mechanical wounding or exposure to GLV released from cut grass, activated a *Lt* 46 kDa mitogen-activated protein kinase (MAPK) within 3 min and a 44 kDa MAPK within 15–20 min in the model grass species *Lolium temulentum* (*Lt*). Currently very little is known concerning the perception, signaling or molecular responses associated with wound stress in grasses. Since GLV are released during wounding, we wanted to investigate what genes and signaling pathways would be induced in undamaged plants exposed to GLV.

**Results:**

RNA-Seq generated transcriptome of *Lolium* plants exposed to GLV identified 4308 up- and 2794 down-regulated distinct differentially-expressed sequences (DES). Gene Ontology analysis revealed a strong emphasis on signaling, response to stimulus and stress related categories. Transcription factors and kinases comprise over 13% of the total DES found in the up-regulated dataset. The analysis showed a strong initial burst within the first hour of GLV exposure with over 60% of the up-regulated DES being induced. Specifically sequences annotated for enzymes involved in the biosynthesis of jasmonic acid and other plant hormones, mitogen-activated protein kinases and WRKY transcription factors were identified. Interestingly, eleven DES for ferric reductase oxidase, an enzyme involved in iron uptake and transport, were exclusively found in the down-regulated dataset. Twelve DES of interest were selected for qRT-PCR analysis; all displayed a rapid induction one hour after GLV exposure and were also strongly induced by mechanical wounding.

**Conclusion:**

The information gained from the analysis of this transcriptome and previous studies suggests that GLV released from cut grasses transiently primes an undamaged plant’s wound stress pathways for potential oncoming damage, and may have a dual role for inter- as well as intra-plant signaling.

**Electronic supplementary material:**

The online version of this article (10.1186/s12870-019-1799-6) contains supplementary material, which is available to authorized users.

## Introduction

Damage to plant tissues due to feeding insects, the grazing of animals, plants crushed by being tread upon or by being mechanically cut is a major form of stress that all plants must endure. Wound stress responses are well characterized in dicot plants. The molecular characterization of wounding was first described in tomato [1] and has been extensively studied in both tomato and *Arabidopsis* [2–4]. Plants respond to mechanical wounding or herbivorous insect attack through the perception of a diverse array of signals, which in turn activate complex signal transduction networks resulting in changes in gene expression. These changes in gene expression alter the plant’s physiology and metabolism. They induce the synthesis of a wide array of defense-related proteins and compounds to mediate the plant’s response at the wound site and systemically throughout the distal portions of the plant [2–5]. The damage inflicted by wounding is perceived locally through damage associated plant-derived signals, or elicitors derived from herbivore-secretions, and systemically by hydraulic, electrical and/or chemical signals [2, 6–11]. In addition to proteins such as kinases, receptors, phospholipases, GTPases, ion channels, NADPH oxidase, calmodulin, calcium binding proteins and transcription factors utilized by the signaling pathways, plants use a variety of small molecules such as jasmonic acid (JA) derivatives, salicylic acid (SA), ethylene, reactive oxygen species (ROS) and calcium to mediate responses to wounding [2, 6–16]. Hydrogen peroxide has also been shown to function as an essential component of systemic wound signaling in *Arabidopsis* [17]. At the core of wound signaling is the JA biosynthetic pathway and its bioactive derivatives, which interact with a complex of interacting proteins, which regulate the expression of JA-responsive genes [2, 3, 9, 18–20]. The JA family of oxylipins are key signaling molecules that mediate the plant’s response to wounding [9] and have also been shown to play a significant role in many aspects of growth, development, and environmental responses in plants [9, 21]. Jasmonate biosynthesis originates in the chloroplast through the release and conversion of polyunsaturated fatty acids. The release of these fatty acids is through the action of lipases, more specifically linolenic acid by phospholipase A (PLA) [21, 22]. These long-chain fatty acids are then oxidized through lipoxygenase-catalyzed reactions and are converted to 13-hydroperoxy derivatives. These fatty acid hydroperoxides are subsequently converted by allene oxide synthase (AOS) and allene oxide cyclase (AOC) and other enzymes to JA [21, 22].

There are also JA-independent wound signaling pathways that control the expression of distinct sets of target genes or that modulate the expression of JA-responsive genes [9, 23–25]. Furthermore, there appear to be some differences between gene expression patterns associated with mechanical wounding and those produced by insect feeding [26, 27]. While there is considerable overlap, there are transcriptional responses that appear to be specific to insect feeding or the application of insect oral secretions to wound sites [8, 9, 28]. This complex network of interacting signals and pathways provides an avenue for the plant to redirect resources to recover from damage and produce a wide range of defense related proteins and compounds to protect it from further herbivore attack.

Another class of signaling molecules that are produced and released in response to wounding stress are volatile compounds [29, 30]. Over 1700 volatile compounds have been identified in plants and are mainly represented by terpenoids, phenylpropanoids/benzenoids, fatty acid and amino acid derivatives [29, 31]. During wounding, the released volatiles are comprised of a complex blend of compounds with distinct chemical signatures that can differ in their composition as a result of damage to plant tissues from herbivores feeding or mechanical wounding [32–38]. Plant volatiles have been shown to play a role in plant defense against insects and pathogens [31–39] and have been implicated in plant responses to different abiotic stresses [30, 40, 41]. Furthermore plant volatiles may prime or enhance the plant’s response to particular stresses and this priming effect may occur via inter- and/or intra-plant signaling [36, 38, 39, 42–50]. When grasses are mechanically damaged they release a volatile chemical blend comprised of 6-carbon compounds that include aldehydes, esters, and alcohols called green leaf volatiles (GLV) [51, 52]. These GLV are synthesized through the hydroxyperoxide lyase branch of the oxylipin metabolism pathway [22, 51]. Interestingly, both JA and GLV utilize similar precursors for their biosynthesis [22, 51, 52]. As more research is conducted it is becoming clear that plant volatiles can play a significant role in the wound stress response.

The wound response in monocots has not been as well characterized as in dicots. However, the wound response in *Brachypodium* and cereal grasses such as rice, wheat, barley, and maize appears to utilize many of the same signaling components and activate similar genes that are found in the well-studied dicot systems. Some examples include hydraulic and electrical signals in barley [53, 54], components of the octadecanoid pathway in rice and maize [55–59], rice MAP Kinases [60], wound inducible genes and proteins in rice and maize [61–65], release of volatile organic compounds [28, 66, 67], and wound defense proteins such as proteinase inhibitors [68–70].

Unlike most cereal grasses, forage and turf grasses are repeatedly being cut or grazed upon by animals throughout their lifecycle. While these grasses are fed upon by insects and livestock, mechanical wounding remains one of the major stresses to which grasses are exposed to on a continuing basis. Currently, very little is known concerning the perception, signalling pathways or molecular responses to wounding in forage and turf grasses, and how these responses affect persistence, the regrowth of new tissues and the long-term quality of the grass. Some wound-related signaling components have been identified in forage and turf grasses. A wound-induced oxidative burst was shown in ryegrass leaf blades [71]. Furthermore, it has been shown that mechanical wounding rapidly activated a 46 kDa and a 44 kDa mitogen-activated protein kinase (MAPK) in six different grass species. In the model grass species *Lolium temulentum* (*Lt*), the 46 kDa MAPK was found to be activated within five minutes of wounding, both locally and systemically in an adjacent unwounded tiller. [72]. A transcriptome analysis of sheepgrass comparing defoliation and mechanical wounding identified a significant number of genes and metabolic pathways impacted by these stresses, including genes involved in the biosynthesis of JA. [73].

When forage and turf grasses are cut, they release a cloud of a diverse mixture of chemical compounds, termed GLV, the composition of which has been described for tufted hairgrass [52], into the surrounding atmosphere. Depending on atmospheric conditions these compounds can travel great distances. Furthermore it was reported that after cutting leaves and stems in grass and clover, this initial burst of GLV emissions was followed by a second more intense emission lasting for several hours as the cut vegetation dried out [74]. Therefore, this GLV exposure to neighboring plants can last for hours, not minutes after cutting. Interestingly in Arabidopsis, intermittent exposure to trace amounts of GLV emitted by freshly damaged plants over a period of 3 weeks induced defense related responses in undamaged neighboring plants [47, 75]. Currently very little is known concerning the molecular responses of exposure to GLV in adjacent or neighboring undamaged grasses. Recently it has been shown that just one-minute of exposure to GLV released from cut grasses was enough to activate the *Lt* 46 kDa MAPK within three minutes and the *Lt* 44 kDa MAPK within 15 min [76, 77]. This activation pattern showed similar kinetics to those observed after wounding and the GLV induced MAPK bands displayed nearly identical migration on SDS-PAGE gels to the wound activated MAPK bands. Thirteen different commercially available plant volatile compounds, which included alcohols, aldehydes and ketones were tested and shown to activate these same *Lt* MAPKs [77]. Furthermore, GLV derived from three other grass species as well as tomato, a dicot, were also shown to activate these MAPKs in *Lt*. These results suggest that the perception and the activation of these MAPKs are not specific to volatiles released from a specific plant species or to a specific compound within the GLV mixture, but rather are a generalized response to the release of plant volatiles associated with plant damage in its environment.

In this study we extend our investigation into the response of grasses to GLV and its connection to the wound response. The model grass *Lt* was exposed to GLV and an RNA-Seq transcriptome was generated in order to identify genes, metabolic and signaling pathways that were induced. The analysis of this GLV-induced *Lt* transcriptome revealed differentially expressed sequences (DES) involved in the JA and phytohormone biosynthesis, and proteins associated with growth and stress related pathways. This research provides new insights into molecular mechanisms utilized by grasses in response to wounding.

## Methods

### Plant materials

*Lolium temulentum* L. (*Lt*, Darnel ryegrass) cv. Ceres seeds were planted in 36 well flats, 5 cm × 3.8 cm × 5 cm well (1 plant/well) or 5 plants per pot TSD4 Square Height: 8.8 cm × 8.8 cm × 10 cm Volume: 540 ml (McConkey Co.) containing Sun Gro Professional MM840 PC RSi (Sun Gro Horticulture, Canada). Plants were grown under 12 h (RNA-Seq plants) or 14 h (qPCR plants) photo-periods at 23 °C day and 18 °C night in Conviron E15, PGR15, PGV36 or PDW40 (Conviron, Winnipeg, Canada) growth chambers. Plants were fertilized weekly using Technigro 20–18-20 all-purpose fertilizer (Sun Gro Horticulture, Canada). *Lolium temulentum* cv. Ceres seed was originally obtained from Dr. Lloyd T Evans (CSIRO, Canberra Australia) and all experiments were conducted using seeds increased from this original seed.

### Plant treatments

#### GLV RNA-Seq transcriptome

*Lt* leaf cuttings (3–6 cm length) were crushed by hand and placed on the bottom of 25 cm height X 23 cm diameter clear polycarbonate cylinders (Nalgene) to a depth of approximately 2–3 cm and fitted with non air-tight lids. These cylinders with the grass clippings were then placed into a growth chamber under light and allowed to out gas for 5 min. 3–4 week-old *Lt* plants (2–3 tiller stage) were then placed into the cylinders, which were then closed with lids. The plants are incubated/exposed to GLV for 1 h in the cylinders. After 1 h all the plants are removed from the cylinders (1 h time point) and the cylinders were removed from growth chamber. The plants remained in the chamber for the remainder of the time course. Time points collected: 1 h = 1 h GLV exposure (G1); 2 h = 1 h GLV exposure + 1 h post exposure (G2); 6 h = 1 h GLV exposure + 5 h post exposure (G6). Three plants were collected / time point, a total of 3 biological replicates for each treatment and time point. The aerial portions of the plant plus root crown were collected, tissue was placed in foil packets, quick frozen in liquid nitrogen and stored at − 80 °C until processed. The untreated control (U) plants were treated identically as GLV exposed plants except there was no GLV exposure; they were treated in a different growth chamber. The control samples were collected at the same time as the GLV exposed plants (1 h = U1; 2 h = U2; 6 h = U6).

#### GLV qPCR samples

GLV exposure was as described above: Time points collected, 0 (no treatment), 1, 2, 4, 6 and 12 h. Five plants were collected / time point. Tissue collection was performed by removing and discarding the upper leaf material. The tissue collected for analysis contained only the root crown and the remaining 3–9 cm of pseudo-stem/leaf tissue per tissue sample. The collected tissue samples were placed into foil packets, quick frozen in liquid nitrogen and stored at − 80 °C until processed. The rational for the removal of the upper portions of the leaf of the plant tissue collected for the GLV samples were so that the analysis would be from the same tissue that would be analyzed for the wounding treatment.

#### Wounding qPCR samples

Wounding of plants was performed by using a pair of pliers to pinch off sections of the tillers (3–4 times), starting from the top of the tiller and following down the leaf blades until you reach about 3–9 cm above the root crown. Time points collected: 0 (no treatment), 1, 2, 4, 6 and 12 h post-wound. Five plants were collected / time point. The remaining aerial portion (3–9 cm of pseudo-stem/leaf) of the plant plus the root crown are collected, placed into foil packets, quick frozen in liquid nitrogen and stored at − 80 °C until processed.

The untreated control plants were treated identically to GLV exposed plants except there was no GLV exposure and they were treated in different growth chambers. Note: only 3–9 cm of pseudo-stem/leaf and the root crown was collected for analysis from unwounded control plants. This was to ensure similar tissues were being analyzed for the control samples when compared to the GLV exposed and wounded plants described above.

#### MAP kinase assay

The wounding and GLV exposure (5 min) of *Lt* plants were performed as previously described [76, 77]. Sample preparation and immunoblot analyses were performed as described previously [76–78].

#### RNA sample preparation and Illumina sequencing

Total RNA from 18 library prep samples for GLV and untreated controls (6 time points × 3 biological replicates each) and 21 qRT-PCR samples (21 treatment/time points) was extracted using Trizol (Invitrogen, Carlsbad, CA), according to the manufacturer’s instructions. RNA concentration and quality were measured using a NanoDrop ND-1000 spectrophotometer (NanoDrop Technologies, Wilmington, DE). RNA quality was also assessed by agarose gel electrophoresis. Samples were treated with DNase using the Turbo DNA-free kit (Ambion, Austin, TX) as directed in the manufacturer’s protocol, with the following exception: instead of final treatment with DNAse deactivation solution, the samples were immediately purified using RNEasy MinElute kit (Qiagen, Germantown, MD) according to the manufacturer’s instructions. Samples were again assessed for quality and quantity as above. RNA qRT-PCR samples were further processed for analysis as described below. Library RNA samples were sent to the Oregon State University Center for Genome Research and Biocomputing (OSU CGRB) for preps and sequencing. Samples were prepared using the Wafergen RNA kit. (WaferGen Bio-systems, Fremont, CA). RNA samples were sequenced on the Illumina HiSeq 3000 using a 150 bp paired end run.

#### Transcriptome assembly

RNA-Seq reads from all samples were combined into a *de-novo* Trinity transcriptome assembly [79]. The Trinity assembly was aligned to ryegrass predicted proteins [80] using BLASTX [81]. Trinity assembled transcriptome contigs that matched the predicted ryegrass proteins were condensed based on alignment overlap to remove redundant assembled contigs. Trinity assembled transcriptome contigs that did not align to the ryegrass predicted proteins were self-aligned using BLASTN. Contigs were condensed based on alignment overlap to remove redundant contigs. Assembled transcriptome contigs were functionally annotated using BLASTX alignments against plant proteins in GenBank [82] and UniProt [83]. Protein functions from top hits were assigned as predicted functions to transcriptome contigs. Gene Ontology (GO) was assigned to assembled contigs using UniProt alignments.

#### Transcriptome analysis

Raw sequences were prepared by trimming Illumina adapters and for low quality using Cutadapt (−q 15,10) [84]. Three replicates were sequenced per sample. BWA-MEM [85] was used to align each replicate to the assembled Lolium transcriptome. Alignment files were manipulated with SAMtools [86]. Each pairwise comparison of GLV (G1, G2, and G6) treated time points were compared to control samples (U1, U2 and U6). Sequence reads per assembled transcript were counted and differentially expressed genes were identified using Cuffdiff [87]. Cummerbund [88] was used to visualize differential expression and quality controls. Over represented GO terms were displayed using WEGO 2.0 [89].

#### qRT-PCR analysis of genes of interest

Validation of RNA-Seq results was conducted on 12 selected genes of interest (GOI). Primer pairs were designed using Primer3Web (v. 4.1.0) (Additional file 1: Table S1). One ug of each sample (described above) was reverse-transcribed to cDNA using iScript cDNA Synthesis Kit (BioRad, Hercules, CA) according to the manufacturer’s instructions. Primer pairs were assessed for amplification efficiency using a standard curve with (5) 1:3 dilutions of the PCR product amplified by the primers (with one cDNA sample as template). Only primers with efficiencies between 90 and 105% were used in analysis. Sample expression was normalized using the previously-analyzed reference genes eurkaryotic elongation factor 1-α (eE1 α) and Ubiquitin5 (UBQ5) [90]. Triplicate reactions were run in 20 ul volumes with 10 ul 2X SSO Universal SYBR Green Supermix (BioRad), 12 picomoles each primer, 1 ul cDNA, and 6.6 ul nuclease-free water. Samples were set up in an ‘all-samples’ configuration, with one primer-pair per plate and all samples run on one plate for each gene. qRT-PCR was run on a BioRad CFX 96-touch with the following parameters: 95 °C for 30 s, followed by 40 cycles of 95 °C for 15 s, annealing/extending temperature between 57 and 59 °C for 1 min. A melt curve from 65 °C to 95 °C followed to ensure single-product amplification. No-template controls (NTC) were run for each primer pair. No NTC samples had Cq values below 36.5 and most were undetected.

#### qRT-PCR data analysis

Data were analyzed using BioRad CFX Maestro 1.0 software. Plates were combined in a Gene Study, with one plate each for a GOI and the two reference genes. Actual primer efficiencies were entered before running analysis. Normalized expression (ΔΔ Cq) of each sample was compared to a control sample, Control 0, and analyzed for significant differences at *p* = .05. The primers sets for these DES are listed in Additional file 1: Table S1.

## Results

**Fig. 1:**
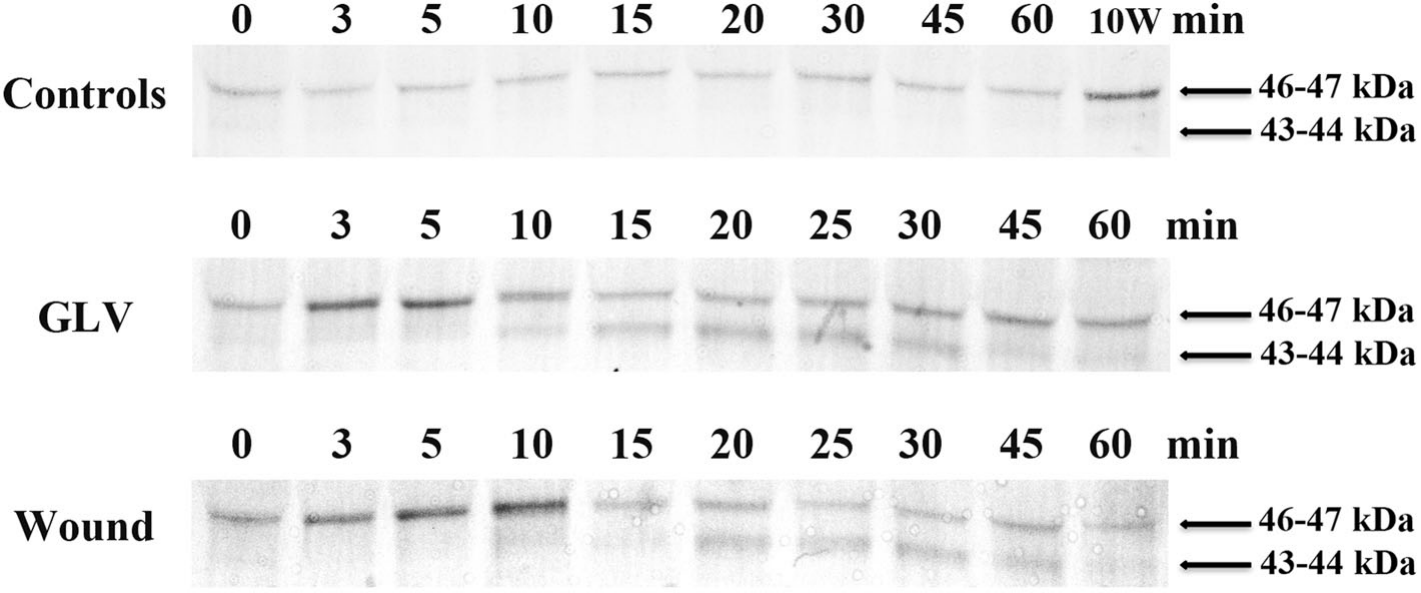
MAPK activation by GLV and wounding . Three to four week-old plants were either exposed to GLV for 1 h or wounded with a hemostat either once or 3 times across all tillers. The entire aerial-portion of the plant (one plant/tiller per time point) was collected over the course of 1 h at the times indicated. 10 W: 10 min wound sample. To determine MAPK activity, immunoblots of protein extracts were performed using anti-phospho-MAPK (Erk1/2) antibody. All experiments were repeated a minimum of three times using samples from independent experiments. Representative blots are presented

*Lolium temulentum L.* (*Lt*, Darnel ryegrass) is a diploid self-fertile model forage/turf grass species [91]. Previously we have shown that mechanical wounding and GLV released from cut grass activated a 46 kDa MAPK within 3 min and 44 kDa MAPK within 20 min after wounding or exposure to GLVs in *Lt* [72, 76, 77] (see Fig. 1). Since GLV rapidly activated MAPK signaling cascades, we wanted to examine what early genes were being activated as a result of GLV exposure. Sequencing produced from 15.7 M to 21.8 M reads per sample. The initial read counts and percent alignments for expression libraries generated for *Lt* plants after exposure to the GLV are summarized in Additional file 2: Table S2. Generation of the *Lolium* GLV transcriptome yielded 207,887 contigs with an average length of 562.89 bp; and after removing isoforms yielded a total of 159,201 unique/distinct sequences as described in Additional file 3: Table S3.

Results of GLV treatments were evaluated using differential expression of fragments per kilobase million (FPKM) values between the GLV exposed and their corresponding untreated controls for 1, 2 and 6 h time points. If FPKM was 0 then it was converted to 0.05. Fold change values were converted to log2 and filtered by selecting FPKM log2 values ≥ ±1, pvalue ≤0.1, and false discovery rate (FDR) ≤ 0.5, and separated into up and down-regulated data sets.

These differentially expressed sequences (DES) were then annotated and gene ontology (GO) enrichment analyses was performed. With no reference genome available for forage or turf related grasses, using existing databases for other plant species resulted in annotation of 87.5% of the total DES identified. Additional file 4: Table S4, contains identified nucleotide sequences, annotation, Reads Per Kilobase Million and log_2_ values for GLV treated samples and their corresponding untreated controls, *p*-values, FDR and GO terms are on separate spreadsheets for the up- or down-regulated DES at each time point.

Analysis of the total DES showed a strong initial burst within the first hour of GLV exposure containing 60.7% (2947) of the total combined DES in the up-regulated dataset. This total was reduced by almost half to 31.2% (1514) DES 2 h post exposure, and after 6 h only 8.1% (395) of the DES were found to be up-regulated. In contrast, the percentage of down-regulated DES remained constant over the first 2 h post exposure with 45.7% (1319) DES after 1 h and 42.3% (1223) DES 2 h post exposure and dropping off to just 12% (346) DES after 6 h.

**Fig. 2:**
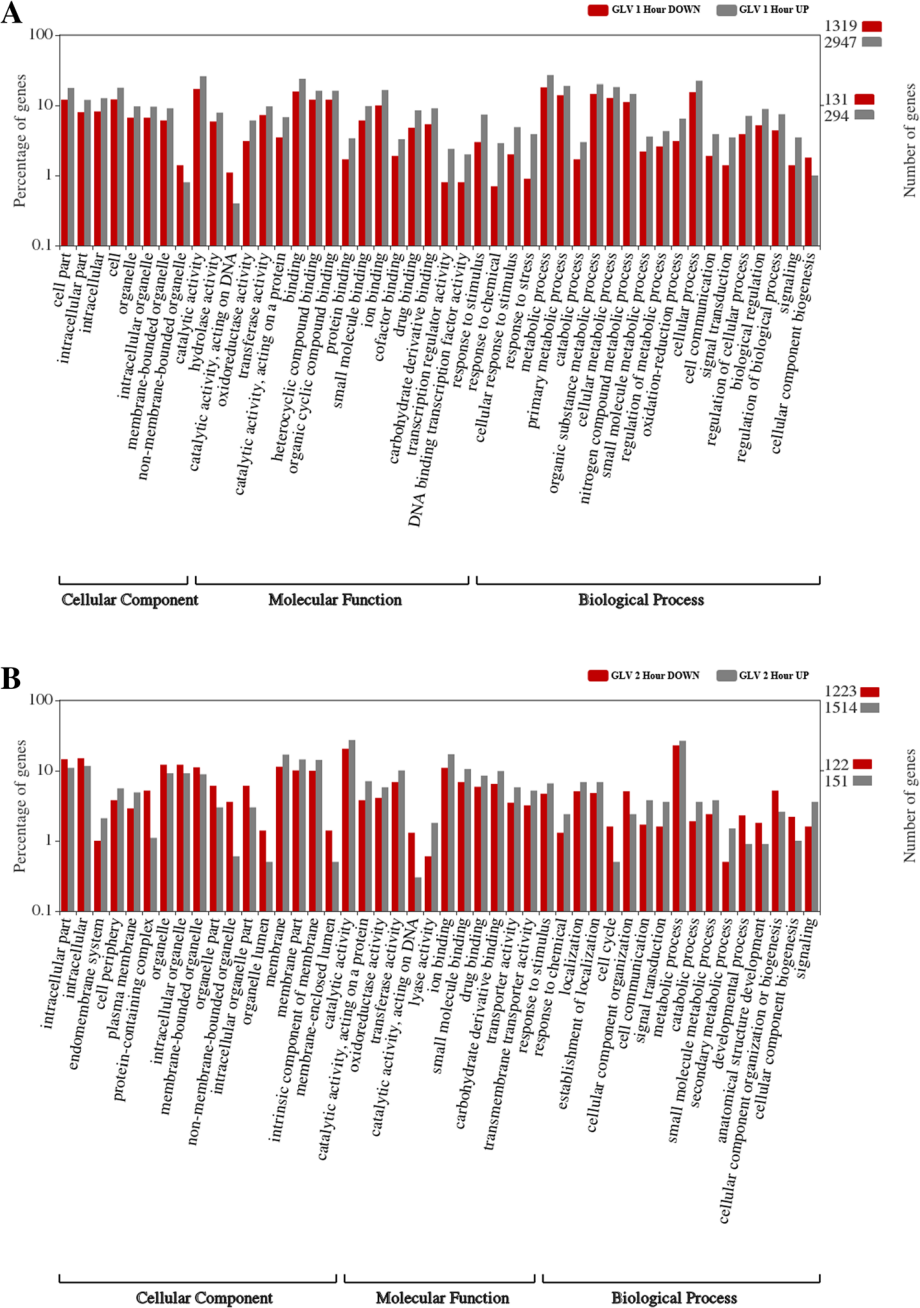
GO Analysis for up-and down regulated of GLV databases (**a**) one h after exposure and (**b**) two h after exposure. Red bars = percentage down-regulated DES; Gray bars = percentage up-regulated DES. Numbers on the right hand axis represents the percentage of genes in log_10_ scale. Numbers on the left axis is the number of total DES contained in up-regulated (gray) and the down-regulated (red) datasets used in analysis

In order to derive functional information from the DES datasets we performed Gene Ontology (GO) enrichment analyses. Figure 2 shows GO classification results of the DES for 1 and 2 h post GLV exposure. The DES 6 h post GLV exposure GO analysis was not informative due to the small number of DES and is not shown. The GO analyses were separated into three categories: cellular component, molecular function, and biological process. The lack of a reference genome for forage/turf grasses limits our ability to gain significant insight relating to the expression levels within a specific category compared to the levels expected in the genome as a whole, therefore we focused our attention on those categories displaying the largest ratio of up- to down-regulated DES as a potential discriminator. One hour after GLV exposure, the GO sub-categories that appear to be most enriched for up-regulated DES are transcription regulator activity, DNA binding transcription factor activity, response to stimulus, response to chemical, cellular response to stimulus, response to stress, oxidation-reduction process, cell communication, signal transduction and signaling. These categories suggest the plant displayed a robust signaling response to GLV exposure within the first hour. The sub-categories that displayed the most enrichment for down–regulated DES after 1 h were non-membrane bound organelle, catalytic activity, acting on DNA and cellular component biogenesis. After 2 h, the most enriched sub-categories for up-regulated DES were, endomembrane system, lyase activity, cell communication, signal transduction, secondary metabolite process and signaling. The 2 h down-regulated DES were reflected in the sub-categories associated with protein-containing complex, non-membrane bound organelle, intracellular organelle part, organelle lumen, membrane enclose lumen, catalytic activity, acting on DNA, cell cycle, developmental process, anatomical structure development, cellular component organizations or biogenesis and cellular component biogenesis.

**Fig. 3:**
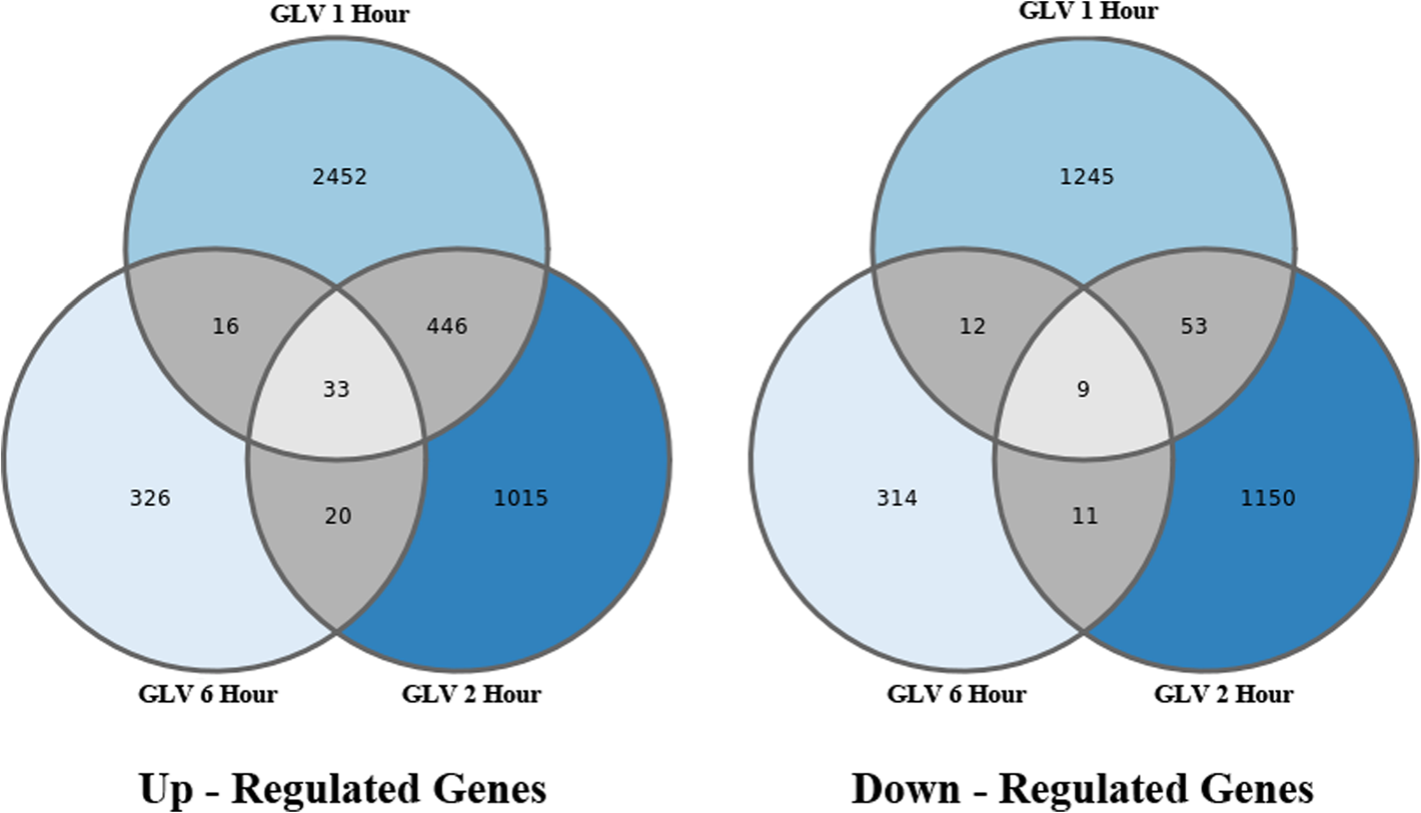
Venn diagrams for the up- and down-regulated DES after 1, 2, and 6 h GLV exposure

In order to investigate the common DES between the up- and down-regulated GLV datasets for 1, 2 and 6 h, we compared gene contigs within each category as visualized in Fig. 3 with Venn diagrams. Of the 4308 up-regulated DES only 0.0076% (33) were found to be up-regulated in all three time points, and only 16% (479) of DES in the 1 h were also up-regulated after 2 h, and 1.6% (49) of the 1 h DES after 6 h. Similar percentages were observed for the down-regulated DES, with only 0.0068% (9) found to be common between the three time points, 4.7% (62) DES in the 1 h were also found in the down-regulated after 2 h and 1.6% (21) of the 1 h DES after 6 h. The up and down-regulated DES corresponding to the time-point comparisons and their values are list in Additional file 5: Table S5. Analysis of the 33 shared up-regulated DES across the three time points (Table 1) found that eight of the DES annotated as lipoxygenases, three for phenylalanine ammonia-lyase, two as polyphenol oxidase, six as hypothetical/uncharacterized proteins, and the rest were uniquely annotated to other genes.

**Table 1:** GLV Up-Regulated DES Common Between 1, 2 and 6 Hours Databases

Contig/Sequence Identifier	Annotation
TRINITY_DN60028_c0_g1_i2	heat shock protein
TRINITY_DN63652_c1_g11_i14	EF-hand calcium-binding protein
TRINITY_DN64086_c2_g2_i1	Subtilisin-like protease
TRINITY_DN65866_c3_g6_i4	Lipoxygenase
TRINITY_DN66129_c1_g2_i1	hypothetical protein
TRINITY_DN66129_c1_g3_i1	hypothetical protein
TRINITY_DN68283_c0_g5_i6	uncharacterized oxidoreductase
TRINITY_DN68616_c0_g1_i2	beta-amylase
TRINITY_DN68786_c0_g3_i4	thiol protease
TRINITY_DN69436_c3_g1_i11	aldo/keto reductase family protein
TRINITY_DN69992_c1_g1_i1	polyphenol oxidase
TRINITY_DN70336_c1_g5_i10	thionin-like protein
TRINITY_DN70543_c1_g1_i7	polyphenol oxidase
TRINITY_DN70971_c2_g1_i1	phenylalanine ammonia lyase
TRINITY_DN71697_c5_g3_i11	Lipoxygenase (chloroplastic)
TRINITY_DN71736_c1_g1_i1	Lipoxygenase (chloroplastic)
TRINITY_DN71769_c4_g4_i1	dehydrin−/LEA group 2-like protein
TRINITY_DN71848_c2_g1_i2	uncharacterized protein
TRINITY_DN71848_c2_g1_i6	uncharacterized protein
TRINITY_DN72031_c1_g3_i7	uncharacterized protein
TRINITY_DN72171_c2_g2_i20	phenylalanine ammonia-lyase
TRINITY_DN72259_c3_g4_i1	Lipoxygenase (chloroplastic)
TRINITY_DN72450_c4_g8_i1	Lipoxygenase (chloroplastic)
TRINITY_DN72487_c3_g1_i2	Lipoxygenase
TRINITY_DN73639_c0_g9_i2	Lipoxygenase (chloroplastic)
TRINITY_DN73676_c0_g8_i4	Protein tas
TRINITY_DN74007_c2_g5_i10	phenylalanine ammonia-lyase
TRINITY_DN75019_c2_g1_i3	phenylalanine ammonia-lyase
TRINITY_DN75383_c1_g3_i1	linoleate 9S-lipoxygenase3
TRINITY_DN77545_c1_g1_i1	MAP kinase kinase kinase
TRINITY_DN78707_c0_g9_i12	Aspartic proteinase
TRINITY_DN7884_c0_g2_i1	uncharacterized protein
TRINITY_DN80379_c0_g2_i1	Lipoxygenase (chloroplastic)

It should be noted that one of the disadvantages of RNA-Seq transcriptome analysis without a reference genome is that it produces sequence fragments with shorter read lengths. The average sequence length of the transcriptome DES was roughly 500–600 bp, which in most cases is an insufficient length to span the entire coding region of most genes. Therefore DES with similar annotation may map to a similar locus, represent different regions of a single gene sequence, have non-overlapping multiple isoforms, or be family members of the same gene or a different gene/loci entirely.

The GO analysis revealed a very strong emphasis on signaling and responses to stimuli after 1 h of exposure to GLV. Using this as a basis, we conducted keyword searches of the compiled up- and down-regulated DES shared across the three time-points to further identify potential biosynthetic, metabolic and signaling pathways or biological functions represented in the transcriptome. The results of these searches are shown in Table 2.

**Table 2:** Biological Functional Analysis of Venn Database

	UP	DOWN
Total DES (1, 2 and 6 h) Unannotated Sequences	4308 (61%)	2794 (39%)
	508 (11.8%)	462 (16.6%)
	Total DES	
Key Word Search	UP	DOWN
Kinase	395	176
Mitogen activated protein/ MAPK	20	4
Phosphatase	54	22
Receptor	265	111
LRR receptor	62	21
Transcription	211	88
Transcription factor	172	77
WRKY	27	7
Heat shock/chaperone	91	20
Protease/proteinase/peptidase	81	39
Chloroplast/chloroplastic	253	220
Membrane	53	22
Channel	31	8
ABC transporter	46	21
Transport	168	90
Transferase	189	132
Reductase	71	47
Synthase	127	67
Oxidase	82	48
Peroxidase	28	13
P450	58	23
Dehydrogenase	53	29
Calcium/calmodulin	64	10
IAA/auxin	30	8
Cytokinin	10	1
Salicylate/salicylic	9	0
Ethylene	44	6
ABA	7	2
Gibberellin	12	1
Lipoxygenase	23	0
Jasmonate/jasmonic	6	0
Lipase	30	15
Phospholipase A	5	0
Hydroperoxide lyase	1	0
Cellulose synthase	15	10
Proline	17	21
glycine betaine/proline transporter	0	4
phenylalanine ammonia-lyase	18	1
Glucanase	11	4
Ferric reductase oxidase	0	11
Expansin	3	11
GTP	9	6
Dehydrin	2	0
Thioredoxin	7	1
Allene oxidase	6	0
Allene cyclase	1	0
aminocyclopropane	5	3

It should be noted that this type of analysis has shortcomings associated with it. For example, enzymes functioning in a biosynthetic or signaling pathways such as aminocyclopropane-1-carboxylate (ACC) oxidase [92] involved in ethylene biosynthesis, will not show up in the search for ethylene. Therefore some of the categories may be under-represented. Similarly, in GO analysis the significance of a category could be under-emphasized due to the absence of GO terms being assigned to some of the contigs/genes in a dataset, or a single gene could be represented in dozens of GO sub-categories potentially over-emphasizing its significance.

The JA family of oxylipins are key signaling molecules that mediate the plant’s response to wounding [9]. Interestingly many of these important enzymes in the biosynthesis of JA that include PLA (5), lipoxygenases (23), AOS (6) and AOC (1) were exclusively found only in the up-regulated DES dataset. In addition, our search of the dataset for jasmonate found six additional DES in the up-regulated DES dataset. Four of these DES were annotated as JAR1, a gene that encodes for an enzyme that conjugates JA to isoleucine, its biologically active form [93]. Surprisingly, only one hydroperoxide lyase DES [22, 51], a key enzyme involved in the production of GLV, was found in the up-regulated 6 h dataset. The presence of these key enzymes involved in the JA biosynthetic pathway in the GLV-induced transcriptome suggests that plants exposed to GLV may be producing and accumulating JA.

Protein kinases are extensively involved in the plant’s responses to stress, growth and development, and play key roles in the transmission of signals and in the regulation of a wide variety of complex cellular processes within the cell [94, 95]. Our search of the annotations in the Venn datasets found that 395 (9.2%) of the DES in the up-regulated and 176 (6.3%) in the down-regulated datasets contained the term kinase. Previously we have shown that MAPKs are rapidly activated in plants exposed to GLV, wounding and a variety of other abiotic stress [72, 76–78]. We wanted to determine if the transcriptome contained these important signaling proteins. Our search identified 20 DES in the up-regulated (5 MAPK, 5 MAPKK and 10 MAPKKK) and 4 DES in the down-regulated datasets. Phosphatases work antagonistically with kinases in regulating many cellular processes and functions, and our search found 54 DES in up-regulated and 22 in the down-regulated datasets. Receptors are key proteins involved in perception of stimuli or molecules, then transmit this information through a variety of signaling molecules, proteins or pathways leading to a specific response within the cell. Analysis of the transcriptome found 265 DES (receptor) in the up-regulated dataset of which 62 were annotated as LRR-receptors [96], and 111 DES in the down-regulated dataset, of which 21 were LRR-receptors. Furthermore, 89.4% of total DES receptors identified in the up-regulated dataset were also kinases. Another key protein-mediating gene activation within the cell is transcription factors; 172 DES were found in the up-regulated and 77 DES in the down-regulated datasets. Furthermore, WRKY transcription factors are one of the largest families of transcriptional regulators in plants, they have been shown to modulate many plant processes, including the responses to biotic and abiotic stresses. Of the 172 transcription factors found in the up-regulated DES 15.7% (27) were found to be members of this family.

Proteinases and heat shock/chaperone related proteins also play important roles in mediating and regulating many molecular processes within the cell during growth and in response to stress [97–99]. Our search found 91 heat shock related DES and 81 proteases in the up-regulated and 20 and 39 DES in the down-regulated datasets, respectively.

The chloroplast is an important organelle that carries out photosynthesis, producing energy and building blocks for plant growth and development, and is also involved in fatty acid synthesis, amino acid synthesis, and defense responses in plants. Our query found that 5.88% (253) of the up-regulated DES and 7.87% (220) of the down-regulated contained the word “chloroplast” within their annotations, indicating that GLV exposure significantly altered expression of genes associated with this important organelle.

Calcium plays a key role in many signal transduction pathways in plants, is crucial for the plant’s response to various stresses and has been shown to have a fundamental role in growth and development [10, 100, 101]. Interestingly the proportion of annotations containing “calcium” was significantly higher at 1.54% (64) in the up-regulated DES dataset and only 0.0036% (10) in the down-regulated dataset. Other signaling molecules and hormones play significant roles in mediating the plant’s response to stress, and Table 2 shows the number of hits in the datasets with annotations for DES containing the terms auxin, gibberellin, ethylene, ABA, cytokinin and salicylate.

Interestingly, while the JA biosynthetic-related DES were unique to the up-regulated dataset, other DES were found to be disproportionately represented in the down-regulated dataset. Expansins are proteins involved with cell wall loosing and growth [102] and 78.6% (11/14) of them in our transcriptome were found in the down-regulated dataset. In addition, our search identified 55% (21/38) of the proline related DES in the down-regulated dataset, with four DES for glycine betaine/proline transporter [103] found exclusively there. All of the DES annotated for ferric reductase oxidase (11), an important enzyme involve in iron transport and uptake [104], were solely identified in the down-regulated dataset.

**Fig. 4:**
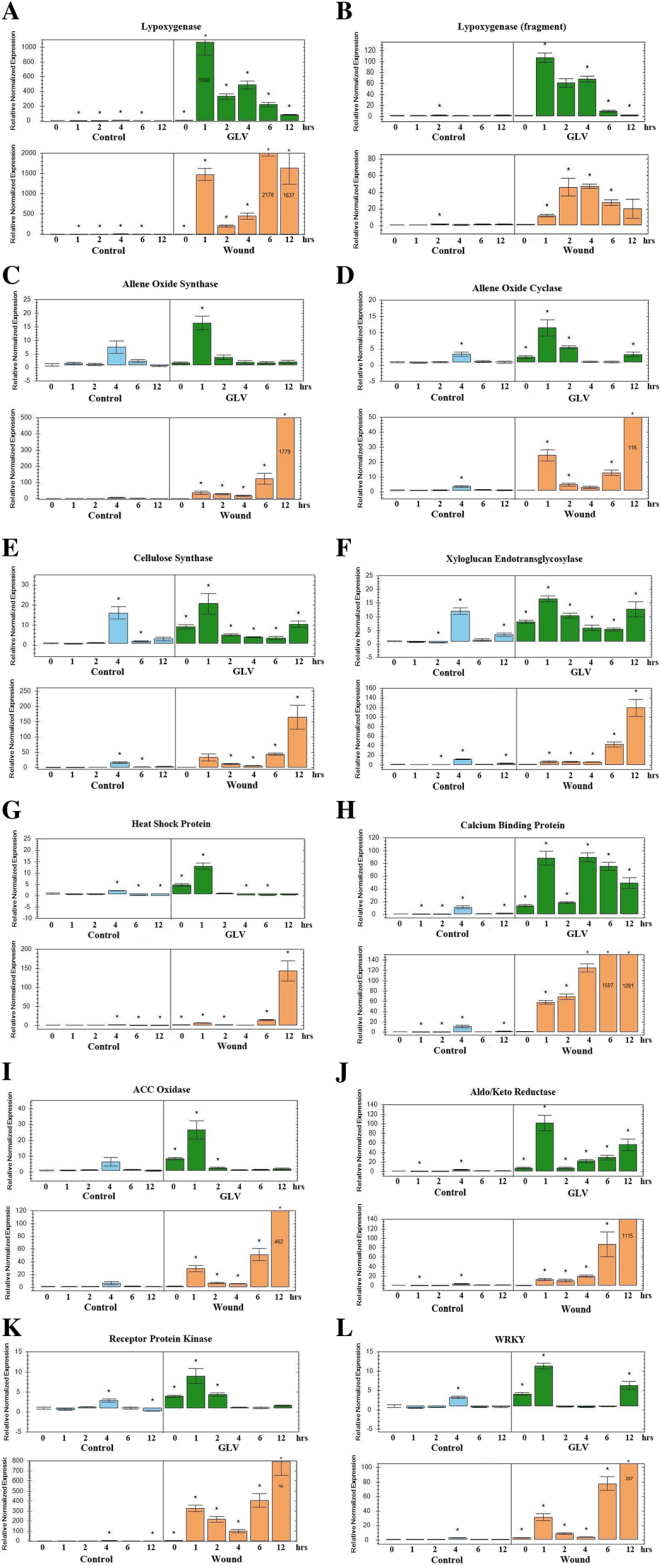
RT-qPCR expression analysis of selected DES after GLV exposure and wounding. Relative expression levels (to control time point 0) of selected DES to 1 h exposure to GLV or after mechanical wounding at the times indicated. **a**) Lipoxygenase (TRINITY_DN75383_c1_g3_i1); **b**) Lipoxygenase (fragment) (TRINITY_DN65866_c3_g6_i4); **c**) Allene oxide synthase (TRINITY_DN74953_c1_g1_i1); **d**) Allene oxide cyclase (TRINITY_DN69828_c0_g2_i50; **e**) Cellulose synthase (TRINITY_DN36463_c0_g1_i1); **f**) Xyloglucan endotransglycosylase (TRINITY_DN58279_c0_g2_i3); **g**) Heat shock (TRINITY_DN64622_c1_g1_i1); **h**) Calcium binding protein (TRINITY_DN63652_c1_g11_i14); **i**) Aminocyclopropane-1-carboxylate (ACC) oxidase (TRINITY_DN69501_c1_g1_i1); **j**) Aldo/keto reductase (TRINITY_DN69436_c3_g1_i11); **K)** Receptor protein kinase (TRINITY_DN68100_c2_g4_i5); **L)** WRKY (TRINITY_DN77648_c2_g5_i14). The primers sets for these DES are listed in Additional File 1:Table S1

In order to validate the transcriptome sequencing results, 12 genes of interest were selected for qRT-PCR analysis. In addition to examining the induction of these genes by GLV, this analysis also investigated their potential induction as a result of mechanical wounding. Since JA has been shown to play a key role in wound signaling in other plant species, four DES coding for potential enzymes involved in JA biosynthesis were chosen for expression analysis. Two lipoxygenases were selected from the eight DES listed in Table 1 containing the common up-regulated DES between 1, 2, and 6 h. AOS and AOC, the two committed enzymes of the JA biosynthetic pathway were also analyzed. Cellulose synthase and xyloglucan endotransglycosylase were selected since they are enzymes involved in cell walls and growth [105]. In our analysis we identified 91 heat shock related DES and 27 WRKY DES (Table 2) in the up-regulated dataset; one was randomly selected from each group for further analysis. The calcium binding protein and the aldo/keto reductase DES were also listed in Table 1. The ACC oxidase DES was selected since this enzyme is involved in ethylene biosynthesis and a receptor protein kinase was randomly chosen from the dataset [92]. The qRT-PCR results of the expression levels of these DES are presented in Fig. 4. All of the selected DES analyzed displayed a rapid induction 1 h after GLV exposure and were also induced by wounding. Interestingly, all the DES except one of the lipoxygenases (Fig. 4B) displayed a very high relative level of expression 12 h after wounding. It should be noted that the 4 h control sample consistently yielded an elevated level of expression regardless of the DES tested. This may indicate an artifact, for instance contamination during the sample preparation.

## Discussion

Forage and turf grasses are challenged by a variety of stresses on any given day, but one of the major stresses these grasses face is mechanical wounding. Since GLV are rapidly released upon wounding and have been shown to rapidly activate MAPK signaling cascades, we wanted to investigate the early molecular responses of grasses after being exposed to GLV and its potential connection to the wound response. Therefore we performed RNA-Seq analysis to identify genes differentially regulated and molecular processes affected after exposure to GLV in the model grass *Lt*.

One of the main findings of this GLV induced transcriptome analysis is that it supports the concept of plant-to-plant communication and GLV induced priming for the wound response in forage and turf related grasses. In an elicitor induced priming response, you would expect to find an increase in the relative level of signaling and transcriptional regulating proteins within the cell prior to the onset of the stress [106–108]. Then when stress occurs, the plant is able to respond more rapidly and robustly to mobilize its resources to meet the oncoming threat or stress. This up-regulation of signaling and transcriptional regulating components would also be transient in nature. The DES datasets and GO analysis of the GLV-induced transcriptome revealed an abundance of DES involved in signaling and response to stimuli/stress within the first 2 h after GLV exposure. The analysis of the datasets found that over 60% of the up-regulated DES were observed within the first hour of exposure and only 8.1% were observed six hours after exposure. Kinases and transcription factors alone comprise over 13% of the total DES found in the up-regulated datasets. With high levels of transcription you would also expect to see an increase in the level of DES coding for proteins that facilitate proper folding and stabilization of newly translated proteins within the cell. As expected, our analysis found an over abundance of heat shock and chaperone proteins in the up-regulated datasets. Furthermore, if GLV priming is occurring then you would expect to see very different patterns of expression for genes in response to wounding and GLV. Genes induced by transient exposure to GLV would display a strong initial induction within the first couple hours, with their expression decreasing over time. However upon wounding, which causes lasting damage to the plant, you would expect to observe an initial induction that increases over time. The expression analysis of the 12 selected DES shown in Fig. 4 appeared to display these types of expression patterns overall. All DES induced by GLV showed a strong initial burst of expression within the first hour of exposure that was significantly reduced after two hours. However in a number of DES, a secondary but reduced induction occurred after 12 h, whereas the DES for calcium binding protein (Fig. 4H) displayed a strong second burst of induction after 4 h that decreased slowly over the remaining eight hours of the time course. In contrast, all DES induced by wounding displayed an induction after the first hour post wounding that increased over time, with their levels of expression peaking at six to twelve hours post wounding. Interestingly, the six–twelve hour delay of maximum expression for many of the DES post wounding implies that the plant is acting like it is in state of shock after a considerable loss of tissue, and its needs to recover before mounting a full response. This data would seem to support the concept of priming by GLV. It will be interesting to see if pre-exposure to GLV prior to wounding will alter the expression patterns of these DES as well as others identified in our transcriptome.

JA has been shown to play a critical role in the wound response in many different plant species [9]. Little is known concerning its role in the wound response in forage and turf grasses. The presence of up-regulated DES in our transcriptome for enzymes in the biosynthetic JA pathway and their induction in our expression analysis (Fig. 3) would suggest the potential production and accumulation of JA in the GLV exposed grass plants. This is the first report that we are aware of showing GLV induction of JA biosynthetic enzymes in forage and turf related grasses in response to wound stress. However this is not surprising, since it has been previously shown that maize plants exposed to GLV resulted in the immediate accumulation of JA that subsequently enhanced JA production and JA-signaling responses upon insect herbivore attack [36]. In addition, when Arabidopsis plants were pretreated with GLV, it enhanced their response to methyl jasmonate (MJ) [109]. GLVs have also been shown to interact with JA and other phytohormones, which influences the outcome of the plant’s defense response against pathogens [13, 35, 37, 110]. However unlike GLV, MJ, the volatile bioactive form of JA, does not activate *Lt* MAPKs [72]. However, pretreatment of plants with MJ prior to wounding did accelerate and potentiate the wound activation of the *Lt* 44 kDa MAPK [72]. This result suggests that MJ specifically enhances *Lt* 44 kDa MAPK activity through an unknown mechanism. This may indicate that while JA may play a significant role in mediating the wound response in grasses and amplifying the overall response, it does not appear to be involved in early stages of signal transduction or perception.

Interestingly, the biosynthesis of both JA and GLV are initially synthesized from the same oxygenated fatty acid precursor, 13(S)-hydroperoxy octadecatrienoic acid (13-HOPT). The 13-HOPT precursor is subsequently converted to JA by the AOS branch of the oxylipin pathway, while GLV are synthesized through the hydroperoxide lyase branch [22, 51]. Surprisingly, our analysis only identified one weakly induced hydroperoxide lyase at the 6 h time point. This coupled with the presence of DES coding for AOS and AOC enzymes and their induction in response to GLV (Fig. 4C and D) indicates that plants exposed to GLV produce JA but not GLV.

In addition to JA biosynthetic DES found in the GLV induced transcriptome, DES coding for enzymes involved in the biosynthesis of other phytohormones such as ethylene and gibberellins, which are known to modulate responses to stress and plant growth, were also found in the up-regulated dataset. The up-regulation of DES for the biosynthesis of phytohormones may indicate alterations in growth potential, as the plant prepares to respond to the potential damage and loss of tissue. This alteration in growth patterns could be tissue specific, where parts of the plant such as the growth of leaf blades could be reduced, as a way to conserve valuable resources in anticipation of the potential loss of tissue due to cutting of the blades. Other plant tissues, such as the roots or the meristematic tissue of the root crown may have it’s growth stimulated or modified to allow the plant to respond more quickly to replenish tissue such as the leaf blades where the majority of photosynthesis occurs. For instance, we found similar amounts of DES for cellulose synthase in the up- and down-regulated datasets, suggesting that cell wall biosynthesis genes may be up-regulated (Fig. 4E and G) in some tissues, while being down-regulated in others. Similarly 17 DES coding for xyloglucan endotransglucosylase/hydrolase protein [105], a protein involved in cell wall expansion, were found in the up-regulated dataset and 10 DES were found in the down-regulated dataset. Surprisingly, the majority of expansin DES, a protein involved in cell wall loosening, were found in the down–regulated dataset. The action of xyloglucan modifying enzymes and expansins can work together during stress to loosen cell walls allowing for further growth [105]. Therefore, the abundance and complement of proteins involved in cell wall growth could vary depending on the tissue. This may result in different outcomes, such as strengthening the stem or modifying the cell walls in the root crown, increasing its capacity to respond more quickly with increased growth to replenish tissue losses due to wounding. Unfortunately, the transcriptome was generated from the aerial portions of the plant and the root crown and as a result is focused on the global transcriptional profile for the plant as a whole, and is not specific for any given tissue. Therefore, it is difficult to determine the specific effects of GLV on the plant growth by just comparing the ratio and relative abundance of these cell wall modifying enzymes and proteins. However we can conclude, due to their abundance, that there appears to be alterations in the composition of the cell walls within the plant in response to GLV.

In response to any stress, a wide array of protein kinases are utilized to transduce the signal generated by a stimulus that the plant recognizes, eventually leading to gene activation. MAPKs play an integral role in this process. MAPK modules, consisting of three functionally linked kinases, act as important converging nodes for stress signals [111, 112]. They have been shown to play an integral role in wound response [111–113]. Our GLV induced transcriptome contained 20 up-regulated DES annotated as MAPKs that included each type of MAPK in the signaling cascade or module. While we have previously described two distinct MAPKs, which are activated by wounding and GLV exposure and classified by their molecular weight, we have not yet been able to identify or clone them. The MAPKs DES identified in this transcriptome will provide an excellent molecular resource for potentially cloning and identifying MAPKs involved in the wound response in forage and turf grasses. Interestingly, it has been shown in *Arabidopsis* that the increased accumulation of MAPKs is an important step in priming plants for full induction to stress responses [114]. Therefore, the induced MAPK DES in our transcriptome may also play a significant role in priming grasses for wound stress. In addition to kinases, 172 DES coded for transcription factors, 27 of which were identified as members of WRKY family of transcription factors. WRKY proteins function via interactions with a diverse array of protein partners, including MAPK proteins and calmodulin as well as a diverse array of other proteins [115, 116]. The WRKY proteins mediate responses to a wide range of abiotic stress including wounding [115, 116]. It was shown in tobacco that two WRKY transcription factors regulate the expression of JA biosynthesis genes in response to wounding by herbivores [117]. Expression analysis of one of the WRKY DES (Fig. 4L) showed that it was induced not only by GLV exposure but also by wounding, indicating that this WRKY, and potentially other WRKYs identified in the up-regulated database, may play a role in the wound response.

Another significant class of proteins known to play a critical role in perception and signaling are receptors. There was an abundance of DES encoding receptors found in our transcriptome (Table 2). Of the 265 DES found in the up-regulated dataset, over 89% of them were receptor kinases, one of which was shown to be induced by both GLV and wounding (Fig. 4K). Other DES receptors identified were for UVB, glutamate, vacuolar sorting and the hormones gibberellin, abscisic acid and ethylene. With an increase in the synthesis of phytohormones and signaling molecules, we would expect a concurrent increase in the concentration of their receptors and interacting proteins in order to effectively amplify the signal. Therefore in a priming response, increasing the abundance of the all the components of the signaling networks or pathways, including receptors, would increase the diameter of the signaling pipeline. This would reduce potential bottlenecks for transmittance of the signal, so the plant can respond more quickly and robustly to the actual stress.

Calcium and calmodulin proteins play a fundamental role in plant growth and development, as well as key signaling components in the plant’s response to wounding and other stresses [100, 101]. Furthermore, it has been shown that plant volatiles can regulate the activities of calcium-permeable channels and promote cytoplasmic calcium transients in Arabidopsis leaf cells [118]. As expected in response to a plant elicitor that results in a strong induction of genes, we found a significant number of calcium/ calmodulin related DES in our up-regulated dataset. These included calmodulin-binding transcription activators, calcium-dependent protein kinases, calcium-transporting ATPases, calcium channel proteins and a number of calcium-binding proteins, one that displayed very strong induction to GLV and wounding (Fig. 4H).

While other types of signals, such as ROS [10, 15, 17] are used for intra-plant signaling to undamaged systemic tissue, volatile compounds have been implicated to play a more significant role in plant species with less developed vascular systems [49]. The architecture of the grass plant is such that each individual tiller growing from the root crown is like its own individual plant. One can easily clone grass plants by separating the tillers at the root crown and planting them. The poor vascularization between these tillers may inhibit the primary wound signal from rapidly traversing the dense tissue of the root crown to reach the adjacent tillers. Therefore, volatile compounds are excellent candidates to act as intra-plant signaling molecules for wound stress in grasses, since they are released into the surrounding atmosphere almost immediately after the tissue is damaged. They can travel unencumbered to the surrounding tillers where they are initially perceived. This results in the rapid activation of MAPK signaling cascades within minutes. As found in our transcriptome analysis, a wave of transcriptional activity up-regulating a wide array of genes involved in signal transduction occurs within the first hour after exposure, priming the rest of the undamaged tillers for potential damage that may come. However, the plant must distinguish between damage to itself or to its neighbor, so as not to waste valuable resources. While GLV provides one level of signaling and alone can prime undamaged tissues for wound stress, secondary signals such as hydraulic, electrical and ROS, [10, 15, 17] must travel from the damaged tissue through the root crown up into the systemic tiller in order to induce a full and complete response to wound damage.

While this study provides insights into the potential early signaling aspects of the wound response, it fails to shed light on the type of genes that the plant would use for its actual recovery from tissue damage or loss. These genes would only be identified in the transcriptional profiles of a wound-induced transcriptome, most likely 6–24 h post-wounding. The growth and recovery of grass plants can be quite striking. In the green house, it’s not unusual to observe 3–6 cm of growth within the first 24 h after cutting mature grass plants down to about 3 cm above the root crown. The identification of genes involved in recovery and regrowth will provide not only valuable insight into how grasses cope with continued loss of biomass on a regular basis, but could also provide a molecular resource that has the potential to be utilized to develop approaches to improve growth and long-term fitness of grasses in the field. Future research will be directed at elucidating the genes involved in recovery after wounding. It will also be of interest to investigate the priming effect by: (1) analyzing the transcriptional profiles for specific tissues to determine for example, does GLV exposure to only the leaf blades result in alterations to the transcriptional profile in the roots?; (2) does pretreatment with GLV prior to wounding alter the induction intensity and temporal patterns of expression when compared to wounding alone? (3) What differences and similarities will be found when comparing the transcriptional profiles of a wounded tiller and its systemic undamaged tiller to our GLV induced transcriptome described herein? This future research will advance our understanding of the response to wounding in forage and turf grasses.

## Conclusion

The analysis of the GLV- treated *Lt* transcriptome reveals the up-regulation and predominance of a diverse array of signaling related proteins, supporting the concept that GLV are priming the plant for oncoming wound stress. This is also supported by the fact that GLV are released into the air almost immediately after wounding, and are rapidly perceived and responded to within three minutes of exposure through the activation of MAPK signaling cascades. The genes up-regulated in response to GLV exposure are also strongly induced by wounding. Furthermore, a large percentage of the GLV induced genes encode for proteins that are components of signaling networks. These include transcription factors, kinases, calcium associated signaling proteins, receptors, as well as the biosynthetic enzymes for the synthesis of secondary signaling molecules such as JA and phytohormones. The induction of these genes occurs rapidly within the first hour after GLV exposure. They are transiently increased, with their induction subsiding within hours of initial perception of the signal. Collectively these data, along with those from previous studies [72, 76, 77], strongly support that GLV is priming the plant for wounding, but also functions as an intra– as well as inter-plant signal in grasses.

### Limitations

No reference genome exists for forage and turf grass species, which limits the types and depth of analysis that can be performed on the GLV grass transcriptome databases. Roughly 13% of our differentially expressed sequences we not annotated. Furthermore one of the disadvantages of RNA-Seq transcriptome analysis is that it produces sequence fragments with shorter read lengths. Our average sequence length for our DES was roughly 500–600 bp, which in most cases is an insufficient length to span the entire coding region of most genes. Therefore DES with similar annotation may map to a similar locus, represent different regions of a single gene sequence, have non-overlapping multiple isoforms, be family members of the same gene or a different gene/loci entirely. The actual concentrations of GLV in the field that forage or turf grasses are exposed to when they are cut, as in a lawn or for collection of hay, are unknown. However, cut grass will release high levels of GLV that can be detected at great distances suggesting that in localized areas GLV concentrations could approach those used during our experiments.

## Additional files

Additional file 1: **Table S1.** List of primers for selected DES used for qRT-PCR expression analysis. This table contains, DES gene name, qPCR primers, amplicon length, annelling temperatures and efficiency (XLSX 9 kb)

Additional file 2: **Table S2.** Table of sequence read counts and percent alignments, at 1, 2, and 6 h time points and their corresponding untreated controls. (XLSX 45 kb)

Additional file 3: **Table S3.** Table describing the GLV transcriptome values, contig length range and # of unique sequences (XLSX 40 kb)

Additional file 4: **Table S4.** List of DES on spreadsheets for the up- or down-regulated DES at 1, 2, and 6 h time points. These spreadsheets contain the identified nucleotide sequences, annotation, Reads Per Kilobase Million and log_2_ values for GLV treated samples and their corresponding untreated controls, *p*-values, FDR and GO terms are on separate spreadsheets for the up- or down-regulated DES at each time point. (XLSX 2883 kb)

Additional file 5: **Table S5.** List of DES on spreadsheets for the up- or down-regulated DES Venn comparisons at 1, 2, and 6 h time points. These spreadsheets contain the locus designations, annotation and GO terms, and log_2_ values for GLV treated samples and their corresponding untreated controls, for the up- or down-regulated DES for each related group shown in the Venn diagrams (Fig. 3). (XLSX 884 kb)

## Abbreviations

13-HOPT: 13(S)-hydroperoxy octadecatrienoic acid
ACC: Aminocyclopropane-1-carboxylate
AOC: Allene oxide cyclase
AOS: Allene oxidase synthase
DES: Differentially expressed sequences
ERK: Extracellular signal regulated kinase
FDR: False discovery rate
FPKM: Fragments per kilobase million
GLV: Green leaf volatiles
GO: Gene ontology
GOI: Gene of interest
h: Hour
JA: Jasmonic acid
*Lt*: *Lolium temulentum*
MAPK: Mitogen-activated protein kinase
min: Minutes
MJ: Methyl jasmonate
PAGE: Polyacrylamide gel electrophoresis
SDS: Sodium dodecyl sulfate
VOC: Volatile organic compounds

## References

[1] Green TR, Ryan CA (1972). Wound-induced proteinase inhibitor in plant leaves: a possible defense mechanism against insects. Science..

[2] Schilmiller AL, Howe GA (2005). Systemic signaling in the wound response. Curr Opin Plant Biol.

[3] Wasternack C, Stenzel I, Hause B, Hause G, Kutter C, Maucher H, Neumerkel J, Feussner I, Miersch O (2006). The wound response in tomato–role of jasmonic acid. J Plant Physiol.

[4] Cheong YH, Chang HS, Gupta R, Wang X, Zhu T, Luan S (2002). Transcriptional profiling reveals novel interactions between wounding, pathogen, abiotic stress, and hormonal responses in *Arabidopsis*. Plant Physiol.

[5] Mithöfer A, Boland W (2012). Plant defense against herbivores: chemical aspects. Annu Rev Plant Biol.

[6] Maffei ME, Mithöfer A, Boland W (2007). Before gene expression: early events in plant–insect interaction. Trends Plant Sci.

[7] Maffei ME, Mithöfer A, Boland W (2007). Insects feeding on plants: rapid signals and responses preceding the induction of phytochemical release. Phytochemistrys.

[8] Howe GA, Jander G (2008). Plant immunity to insect herbivores. Annu Rev Plant Biol.

[9] Koo AJ, Howe GA (2009). The wound hormone jasmonate. Phytochemistry..

[10] Gilroy S, Białasek M, Suzuki N, Górecka M, Devireddy AR, Karpiński S, Mittler R (2016). ROS, calcium, and electric signals: key mediators of rapid systemic signaling in plants. Plant Physiol.

[11] Farmer EE, Gasperini D, Acosta IF (2014). The squeeze cell hypothesis for the activation of jasmonate synthesis in response to wounding. New Phytol.

[12] Wu J, Baldwin IT (2009). Herbivory induced signalling in plants: perception and action. Plant Cell Environ.

[13] Erb M, Meldau S, Howe GA (2012). Role of phytohormones in insect-specific plant reactions. Trends Plant Sci.

[14] Kerchev PI, Fenton B, Foyer CH, Hancock RD (2012). Plant responses to insect herbivory: interactions between photosynthesis, reactive oxygen species and hormonal signalling pathways. Plant Cell Environ.

[15] Baxter A, Mittler R, Suzuki N (2013). ROS as key players in plant stress signalling. J Exp Bot.

[16] Zebelo SA, Maffei ME (2014). Role of early signalling events in plant–insect interactions. J Exp Bot.

[17] Miller G, Schlauch K, Tam R, Cortes D, Torres MA, Shulaev V, Dangl JL, Mittler R (2009). The plant NADPH oxidase RBOHD mediates rapid systemic signaling in response to diverse stimuli. Sci Signal.

[18] Katsir L, Chung HS, Koo AJ, Howe GA (2008). Jasmonate signaling: a conserved mechanism of hormone sensing. Curr Opin Plant Biol.

[19] Chung HS, Niu Y, Browse J, Howe GA (2009). Top hits in contemporary JAZ: an update on jasmonate signaling. Phytochemistry.

[20] Howe GA, Major IT, Koo AJ (2018). Modularity in jasmonate signaling for multistress resilience. Annu Rev Plant Biol.

[21] Cheong JJ, Do Choi Y (2003). Methyl jasmonate as a vital substance in plants. Trends Genet.

[22] Wasternack C, Feussner I (2018). The oxylipin pathways: biochemistry and function. Annu Rev Plant Biol.

[23] León J, Rojo E, Sánchez-Serrano JJ (2001). Wound signalling in plants. J Exp Bot.

[24] Titarenko E, Rojo E, Leon J, Sanchez-Serrano JJ (1997). Jasmonic acid-dependent and -independent signaling pathways control wound-induced gene activation in *Arabidopsis thaliana*. Plant Physiol.

[25] LeBrasseur ND, MacIntosh GC, Pérez-Amador MA, Saitoh M, Green PJ (2002). Local and systemic wound-induction of RNase and nuclease activities in *Arabidopsis*: RNS1 as a marker for a JA-independent systemic signaling pathway. Plant J.

[26] Reymond P, Weber H, Damond M, Farmer EE (2000). Differential gene expression in response to mechanical wounding and insect feeding in *Arabidopsis*. Plant Cell.

[27] Korth KL, Dixon RA (1997). Evidence for chewing insect-specific molecular events distinct from a general wound response in leaves. Plant Physiol.

[28] Heil M (2009). Damaged-self recognition in plant herbivore defence. Trends Plant Sci.

[29] Dudareva N, Negre F, Nagegowda DA, Orlova I (2006). Plant volatiles: recent advances and future perspectives. Crit Rev Plant Sci.

[30] Loreto F, Schnitzler JP (2010). Abiotic stresses and induced BVOCs. Trends Plant Sci.

[31] Knudsen JT, Gershenzon J. The chemical diversity of floral scent. In: Dudareva N, Pichersky E, editors. Biology of floral scent. Boca Raton, FL:CRC Press; 2006. p. 27–52.

[32] Röse US, Tumlinson JH (2004). Volatiles released from cotton plants in response to *Helicoverpa zea* feeding damage on cotton flower buds. Planta.

[33] Röse US, Tumlinson JH. Systemic induction of volatile release in cotton: how specific is the signal to herbivory? Planta 2005;222(2):327–335. 10.1007/s00425-005-1528-2 15856281

[34] Rodriguez-Saona CR, Frost CJ (2010). New evidence for a multi-functional role of herbivore-induced plant volatiles in defense against herbivores. Plant Signal Behav.

[35] Scala A, Allmann S, Mirabella R, Haring MA, Schuurink RC (2013). Green leaf volatiles: a plant’s multifunctional weapon against herbivores and pathogens. Int J Mol Sci.

[36] Engelberth J, Alborn HT, Schmelz EA, Tumlinson JH (2004). Airborne signals prime plants against insect herbivore attack. Proc Natl Acad Sci U S A.

[37] Scala A, Mirabella R, Mugo C, Matsui K, Haring MA, Schuurink RC (2013). E-2-hexenal promotes susceptibility to *Pseudomonas syringae* by activating jasmonic acid pathways in *Arabidopsis*. Front Plant Sci.

[38] Baldwin IT (2010). Plant volatiles. Curr Biol.

[39] Dong F, Fu X, Watanabe N, Su X, Yang Z (2016). Recent advances in the emission and functions of plant vegetative volatiles. Molecules.

[40] Lee K, Seo PJ (2014). Airborne signals from salt-stressed *Arabidopsis* plants trigger salinity tolerance in neighboring plants. Plant Signal Behav.

[41] Holopainen JK, Gershenzon J (2010). Multiple stress factors and the emission of plant VOCs. Trends Plant Sci.

[42] Ye M, Glauser G, Lou Y, Erb M, Hu L. Molecular dissection of early defense signaling underlying volatile-mediated defense regulation and herbivore resistance in rice. Plant Cell. 2019;31(3):687–98. 10.1105/tpc.18.00569 PMC6482627 30760558

[43] Kessler A, Halitschke R, Diezel C, Baldwin IT (2006). Priming of plant defense responses in nature by airborne signaling between *Artemisia tridentata* and *Nicotiana attenuata*. Oecologia.

[44] Heil M, Bueno JC (2007). Within-plant signaling by volatiles leads to induction and priming of an indirect plant defense in nature. Proc Natl Acad Sci U S A.

[45] Frost CJ, Mescher MC, Carlson JE, De Moraes CM (2008). Plant defense priming against herbivores: getting ready for a different battle. Plant Physiol.

[46] Karban R, Shiojiri K, Huntzinger M, McCall AC (2006). Damage-induced resistance in sagebrush: volatiles are key to intra-and interplant communication. Ecology.

[47] Shiojiri K, Ozawa R, Matsui K, Sabelis MW, Takabayashi J (2012). Intermittent exposure to traces of green leaf volatiles triggers a plant response. Sci Rep.

[48] Ton J, D’alessandro M, Jourdie V, Jakab G, Karlen D, Held M, Mauch-Mani B, Turlings TC (2007). Priming by airborne signals boosts direct and indirect resistance in maize. Plant J.

[49] Frost CJ, Appel HM, Carlson JE, De Moraes CM, Mescher MC, Schultz JC (2007). Within-plant signalling via volatiles overcomes vascular constraints on systemic signalling and primes responses against herbivores. Ecol Lett.

[50] Kim J, Felton GW (2013). Priming of antiherbivore defensive responses in plants. Insect Sci.

[51] Matsui K (2006). Green leaf volatiles: hydroperoxide lyase pathway of oxylipin metabolism. Curr Opin Plant Biol.

[52] Watkins E, Gianfagna TJ, Sun R, Meyer WA (2006). Volatile compounds of tufted hairgrass. Crop Sci.

[53] Zimmermann MR, Maischak H, Mithöfer A, Boland W, Felle HH (2009). System potentials, a novel electrical long-distance apoplastic signal in plants, induced by wounding. Plant Physiol.

[54] Felle HH, Zimmermann MR (2007). Systemic signalling in barley through action potentials. Planta.

[55] Rakwal R, Tamogami S, Agrawal GK, Iwahashi H (2002). Octadecanoid signaling component “burst” in rice (*Oryza sativa* L.) seedling leaves upon wounding by cut and treatment with fungal elicitor chitosan. Biochem Biophys Res Comm..

[56] Agrawal GK, Tamogami S, Han O, Iwahashi H, Rakwal R (2004). Rice octadecanoid pathway. Biochem Biophys Res Comm..

[57] Zhou G, Qi J, Ren N, Cheng J, Erb M, Mao B, Lou Y (2009). Silencing *OsHI-LOX* makes rice more susceptible to chewing herbivores, but enhances resistance to a phloem feeder. Plant J.

[58] Wakuta S, Suzuki E, Saburi W, Matsuura H, Nabeta K, Imai R, Matsui H (2011). OsJAR1 and OsJAR2 are jasmonyl-L-isoleucine synthases involved in wound-and pathogen-induced jasmonic acid signalling. Biochem Biophys Res Comm.

[59] Szczegielniak J, Borkiewicz L, Szurmak B, Lewandowska-Gnatowska E, Statkiewicz M, Klimecka M, Cieśla J, Muszyńska G (2012). Maize calcium-dependent protein kinase (ZmCPK11): local and systemic response to wounding, regulation by touch and components of jasmonate signaling. Physiol Plant.

[60] Cho K, Agrawal GK, Jwa NS, Kubo A, Rakwal R (2009). Rice OsSIPK and its orthologs: a “central master switch” for stress responses. Biochem Biophys Res Comm..

[61] Shen S, Jing Y, Kuang T (2003). Proteomics approach to identify wound-response related proteins from rice leaf sheath. Proteomics..

[62] Lawrence SD, Novak NG (2004). Maize genes induced by herbivory and volicitin. J Chem Ecol.

[63] Zhang F, Zhu L, He G (2004). Differential gene expression in response to brown planthopper feeding in rice. J Plant Physiol.

[64] Kim KM, Cho SK, Shin SH, Kim GT, Lee JH, Oh BJ, Kang KH, Hong JC, Choi JY, Shin JS, Chung YS (2005). Analysis of differentially expressed transcripts of fungal elicitor-and wound-treated wild rice (*Oryza grandiglumis*). J Plant Res.

[65] van Loon LC, Rep M, Pieterse CM (2006). Significance of inducible defense-related proteins in infected plants. Annu Rev Phytopathol.

[66] Schmelz EA, Alborn HT, Tumlinson JH (2001). The influence of intact-plant and excised-leaf bioassay designs on volicitin- and jasmonic acid-induced sesquiterpene volatile release in *Zea mays*. Planta..

[67] Piesik D, Pańka D, Delaney KJ, Skoczek A, Lamparski R, Weaver DK (2011). Cereal crop volatile organic compound induction after mechanical injury, beetle herbivory (*Oulema* spp.), or fungal infection (*Fusarium* spp.). J Plant Physiol.

[68] Tamayo MC, Rufat M, Bravo JM, San Segundo B (2000). Accumulation of a maize proteinase inhibitor in response to wounding and insect feeding, and characterization of its activity toward digestive proteinases of *Spodoptera littoralis* larvae. Planta..

[69] Tiffin P, Gaut BS (2001). Molecular evolution of the wound-induced serine protease inhibitor wip1 in *Zea* and related genera. Mol Biol Evol.

[70] Mur LA, Xu R, Casson SA, Stoddart WM, Routledge AP, Draper J (2004). Characterization of a proteinase inhibitor from *Brachypodium distachyon* suggests the conservation of defence signalling pathways between dicotyledonous plants and grasses. Mol Plant Pathol.

[71] Le Deunff E, Davoine C, Le Dantec C, Billard JP, Huault C (2004). Oxidative burst and expression of germin/oxo genes during wounding of ryegrass leaf blades: comparison with senescence of leaf sheaths. Plant J.

[72] Dombrowski JE, Hind SR, Martin RC, Stratmann JW (2011). Wounding systemically activates a mitogen-activated protein kinase in forage and turf grasses. Plant Sci.

[73] Chen S, Cai Y, Zhang L, Yan X, Cheng L, Qi D, Zhou Q, Li X, Liu G (2014). Transcriptome analysis reveals common and distinct mechanisms for sheepgrass (*Leymus chinensis*) responses to defoliation compared to mechanical wounding. PLoS One.

[74] de Gouw JA, Howard CJ, Custer TG, Fall R (1999). Emissions of volatile organic compounds from cut grass and clover are enhanced during the drying process. Geophy Res Lett.

[75] Ozawa R, Shiojiri K, Matsui K, Takabayashi J. Intermittent exposure to traces of green leaf volatiles triggers the production of (Z)-3-hexen-1-yl acetate and (Z)-3-hexen-1-ol in exposed plants. Plant Signal Behav. 2013;8(11):e27013. 10.4161/psb.27013 PMC4091332 24301200

[76] Dombrowski JE, Martin RC (2014). Green leaf volatiles, fire and nonanoic acid activate MAPkinases in the model grass species *Lolium temulentum*. BMC Res Notes.

[77] Dombrowski JE, Martin RC (2018). Activation of MAP kinases by green leaf volatiles in grasses. BMC Res Notes.

[78] Dombrowski JE, Martin RC (2012). Abiotic stresses activate a MAPkinase in the model grass species *Lolium temulentum*. J Plant Physiol.

[79] Grabherr MG, Haas BJ, Yassour M, Levin JZ, Thompson DA, Amit I, Adiconis X, Fan L, Raychowdhury R, Zeng Q, Chen Z, Mauceli E, Hacohen N, Gnirke A, Rhind N, di Palma F, Birren BW, Nusbaum C, Lindblad-Toh K, Friedman N, Regev A (2011). Full-length transcriptome assembly from RNA-seq data without a reference genome. Nat Biotechnol.

[80] Islam MS, Studer B, Byrne SL, Farrell JD, Panitz F, Bendixen C, Møller IM, Asp T (2013). The genome and transcriptome of perennial ryegrass mitochondria. BMC Genomics.

[81] Altschul SF, Gish W, Miller W, Myers EW, Lipman DJ (1990). Basic local alignment search tool. J Mol Biol.

[82] Clark K, Karsch-Mizrachi I, Lipman DJ, Ostell J, GenBank SEW (2016). Nucleic Acids Res.

[83] The UniProt Consortium (2017). UniProt: the universal protein knowledgebase. Nucleic Acids Res.

[84] Martin M (2011). Cutadapt removes adapter sequences from high-throughput sequencing reads. EMBnet.Journal..

[85] Li H. Aligning sequence reads, clone sequences and assembly contigs with BWA-MEM. 2013;arXiv:1303.3997.

[86] Li H, Handsaker B, Wysoker A, Fennell T, Ruan J, Homer N, Marth G, Abecasis G, Durbin R (2009). The sequence alignment/map format and SAMtools. Bioinformatics.

[87] Trapnell C, Hendrickson DG, Sauvageau M, Goff L, Rinn JL, Pachter L (2013). Differential analysis of gene regulation at transcript resolution with RNA-seq. Nat Biotechnol.

[88] Goff L, Trapnell C, cummeRbund KD (2013). Analysis, exploration, manipulation, and visualization of cufflinks high-throughput sequencing data. R package version.

[89] Ye J, Fang L, Zheng H, Zhang Y, Chen J, Zhang Z, Wang J, Li S, Li R, Bolund L, Wang J (2006). WEGO: a web tool for plotting GO annotations. Nucleic Acids Res.

[90] Dombrowski JE, Martin RC (2009). Evaluation of reference genes for quantitative RT-PCR in *Lolium temulentum* under abiotic stress. Plant Sci.

[91] Evans LT (1958). *Lolium temulentum* L., a long-day plant requiring only one inductive photocycle. Nature.

[92] Bleecker AB, Kende H (2000). Ethylene: a gaseous signal molecule in plants. Annu Rev Cell Devel Biol.

[93] Suza WP, Staswick PE (2008). The role of JAR1 in jasmonoyl-L-isoleucine production during *Arabidopsis* wound response. Planta..

[94] Stone JM, Walker JC (1995). Plant protein kinase families and signal transduction. Plant Physiol.

[95] Dissmeyer N, Schnittger A. The age of protein kinases. Methods Mol Biol. 2011;779:7–52. 10.1007/978-1-61779-264-9_2 21837559

[96] Diévart A, Clark SE (2004). LRR-containing receptors regulating plant development and defense. Development.

[97] Schaller A (2004). A cut above the rest: the regulatory function of plant proteases. Planta.

[98] Al-Whaibi MH (2011). Plant heat-shock proteins: a mini review. J King Saud University-Science.

[99] Wang W, Vinocur B, Shoseyov O, Altman A (2004). Role of plant heat-shock proteins and molecular chaperones in the abiotic stress response. Trends Plant Sci.

[100] Tuteja N, Mahajan S (2007). Calcium signaling network in plants: an overview. Plant Signal Behav.

[101] Batistič O, Kudla J (2012). Analysis of calcium signaling pathways in plants. Biochim Biophys Acta (BBA)-General Subjects.

[102] Cosgrove DJ (2015). Plant expansins: diversity and interactions with plant cell walls. Curr Opin Plant Biol.

[103] Ashraf MF, Foolad M (2007). Roles of glycine betaine and proline in improving plant abiotic stress resistance. Environ Exp Bot.

[104] Jeong J, Connolly EL (2009). Iron uptake mechanisms in plants: functions of the FRO family of ferric reductases. Plant Sci.

[105] Tenhaken R (2015). Cell wall remodeling under abiotic stress. Front Plant Sci.

[106] Conrath U (2011). Molecular aspects of defence priming. Trends Plant Sci.

[107] Pastor V, Luna E, Mauch-Mani B, Ton J, Flors V (2013). Primed plants do not forget. Environ Exper Bot.

[108] Balmer A, Pastor V, Gamir J, Flors V, Mauch-Mani B (2015). The ‘prime-ome’: towards a holistic approach to priming. Trends Plant Sci.

[109] Hirao T, Okazawa A, Harada K, Kobayashi A, Muranaka T, Hirata K (2012). Green leaf volatiles enhance methyl jasmonate response in *Arabidopsis*. J Biosci Bioeng.

[110] Ameye M, Allmann S, Verwaeren J, Smagghe G, Haesaert G, Schuurink RC, Audenaert K. Green leaf volatile production by plants: a meta analysis. New Phytol. 2018;220(3):666–83. 10.1111/nph.14671 28665020

[111] Cristina MS, Petersen M, Mundy J (2010). Mitogen-activated protein kinase signaling in plants. Annu Rev Plant Biol.

[112] Sinha AK, Jaggi M, Raghuram B, Tuteja N (2011). Mitogen-activated protein kinase signaling in plants under abiotic stress. Plant Signal Behav.

[113] Bonaventure G, Baldwin IT (2010). New insights into the early biochemical activation of jasmonic acid biosynthesis in leaves. Plant Signal Behav.

[114] Beckers GJ, Jaskiewicz M, Liu Y, Underwood WR, He SY, Zhang S, Conrath U (2009). Mitogen-activated protein kinases 3 and 6 are required for full priming of stress responses in *Arabidopsis thaliana*. Plant Cell.

[115] Rushton PJ, Somssich IE, Ringler P, Shen QJ (2010). WRKY transcription factors. Trends Plant Sci.

[116] Chen L, Song Y, Li S, Zhang L, Zou C, Yu D (2012). The role of WRKY transcription factors in plant abiotic stresses. Biochim Biophys Acta (BBA)-Gene Regulatory Mechanisms.

[117] Skibbe M, Qu N, Galis I, Baldwin IT (2008). Induced plant defenses in the natural environment: *Nicotiana attenuata* WRKY3 and WRKY6 coordinate responses to herbivory. Plant Cell.

[118] Asai N, Nishioka T, Takabayashi J, Furuichi T (2009). Plant volatiles regulate the activities of Ca2+−permeable channels and promote cytoplasmic calcium transients in *Arabidopsis* leaf cells. Plant Signal Behav.

